# The Effect of Think Aloud on Performance and Brain Oxygenation During Cycling – An Exploratory Study

**DOI:** 10.1177/00315125221104769

**Published:** 2022-05-21

**Authors:** Amy Whitehead, Catharine Montgomery, Laura Swettenham, Nicola J. Robinson

**Affiliations:** 1 School of Sport and Exercise Science, 4589 Liverpool John Moores University, Liverpool, UK; 2 School of Psychology, 4589 Liverpool John Moores University, Liverpool, UK

**Keywords:** think aloud, cortical oxygenation, performance, cycling, cognition

## Abstract

In this study, we aimed to investigate the effect of Think Aloud (TA) on performance in trained and untrained participants, using functional Near Infrared Spectroscopy, during incrementally paced cycling. A mixed design was implemented with cycling expertise (10 untrained vs. 9 trained) as the between groups variable and trial stage (5 stages of increasing effort), and condition (silent vs. TA) as within groups independent variables (IVs). Dependent measures were changes in cortical oxygenation (O_2_Hb) in 12 areas of the prefrontal cortex (PFC) and physiological indicators of percentage heart rate maximum (%HRmax), average power output (APO), peak power output (PPO), rate of perceived exertion (RPE) and blood lactate ([La]b) over time. Trained cyclists had higher APO and significantly higher PPO from stages 2–5, in addition to a greater increase in PPO over the duration of the test (range 168W–480 W vs. 133W–313 W). There were significant main effects of stage on %HRmax, Bla and RPE (*p* < .001), with effect sizes (ήp^2^) ranging from .31 to .97. On average, HRmax%, [La]b and RPE were significantly lower after stage 2 onwards within the TA trial than the silent trial, even though similar power outputs were obtained. Thus, the TA trial elicited a better pacing strategy. There was no main effect of group on changes in O_2_Hb, though O_2_Hb did change as a function of stage in four areas of the PFC, and as a function of condition in one area. In this first study to assess the effects of TA on performance during self-paced cycling, TA did not disrupt performance outcomes at low through to high levels of physical exertion for either untrained or trained participants.

## Introduction

The Think Aloud method (TA) is a form of verbal reporting in which participants are asked to verbalize their thought processes whilst performing a task (Ericsson & Simon, 1980; 1993). Think aloud has been widely employed in research and practice, both in and outside of sport. For example, within medical education, Pottier et al. (2010) used TA to investigate clinical reasoning in medical students and experts. In addition, TA has been used to investigate cognition in chess (Gobet & Charness, 2006), nursing (Aitken & Mardegan, 2000), and scrabble (Tuffiash et al., 2007). More recently, sport researchers have used TA to understand thought processes in golf (Calmeiro & Tenenbaum, 2011; Kaiseler et al., 2012; Whitehead et al., 2016), stress and coping in tennis (Swettenham et al., 2018), thought processes during running (Samson et al., 2017), thought processes over the duration of a time trial in cycling (Whitehead et al., 2018; Massey et al., 2020), and cognitive differences between adolescent and adult performance in Australian rules kicking (Elliott et al., 2020).

Ericsson and Simon (1980, 1993) proposed three levels of TA verbalizations. *Level 1* involves vocalization of task-relevant thoughts already activated in attention as verbal articulations or inner speech. *Level 2* verbalization requires participants to recode visual stimuli, not regularly verbalized, prior to providing verbalization on the task. Verbalizations should reflect stimuli affecting the focus of the participant through the task, such as when a participant who vocalizes stimuli (sight, sound, and smell) within a task. Eccles (2012) indicated that *Level 1* and *Level 2* verbalizations result from conscious thought processing in short-term memory during task execution, such that there is concurrent verbalization during a task or immediately after its completion. Ericsson and Simon (1993) identified a third level of verbalization, which is referred to as *Level 3*, that occurs when the participant starts to explain their thought processes. However, this level requires linking information to earlier thoughts and information therefore involves retrieving information from long term memory. *Level 3* verbalizations are thought to direct the participant’s attention to their procedures, potentially changing the structure of the thought processes. Given the potential intrusive nature of TA, researchers have critiqued its potential to affect performance in cases when the use of TA changes the cognitive processes mediating task performance from cognitive processes under silent control (Fox et al., 2011). In addition, early research found substantial performance differences in between TA use and silent performance conditions (Bower & King, 1967; Davis et al., 1968).

In response to this critique, Fox et al. (2011) compared performance on tasks that involved concurrent verbal reporting and matched silent control conditions. They found that instructing participants to verbalize their thoughts during the task did not alter performance, whereas directing participants to provide explanations for their thoughts (*Level 3* verbalization) improved performance. However, within this meta-analysis by Fox et al., (2011), most tasks were cognitive in nature. More recently, Whitehead et al. (2015) studied golf performance to investigate the effects of different levels of verbalization (*Level 2 or 3*) instructions for high or low skilled golfers. Their results demonstrated that neither *Level 2* nor *3* verbalizations impaired putting performance in comparison to a silent control condition, providing support for using TA to recognize an individual’s cognitive processes during task performance. Although this study provided support for using TA in a self-paced sport such as golf, the effects of its use in endurance sports is less clear, making it important to assess these effects during such endurance activities as cycling, which is the main aim of this study.

Within endurance sports, Think Aloud has been used to understand runners’ attentional focus during their performance (Samson et al., 2017), cyclists’ cognitions during their real-life time-trials (Whitehead et al., 2019), and expertise differences among cyclists in lab-based experiments (Whitehead et al., 2018). More recently Massey et al. (2020), combined TA and eye tracking technology to assess thought processes and gaze behavior in trained and untrained cyclists during a 16.1 km time-trial. Collectively these studies provide some evidence for the viability of TA use for capturing concurrent thought processes during endurance performance. However, no research has yet investigated TA effects on actual performance. Whitehead et al.’s (2018) study investigated the relationship between TA cognitions, pacing strategies and performance on a 16.1 km cycling time-trial. Although this study reported successful TA effects for identifying differences between trained and untrained performers, participants in this study also reported that TA may have negatively influenced their performance due to having to attend simultaneously to the process of TA and the demands of the task. Therefore, further research is needed to understand the effects of TA on performance in endurance sports.

Outside of sport, Pike et al. (2014) conducted a study that measured the effect of TA on workload using functional Near Infrared Spectroscopy (fNIRS). Participants were asked to perform a mathematical task whilst using TA and during a silent trial. Pike et al. (2014) predicted that since TA uses Working Memory (WM) resources, inclusion of spoken protocols might negatively affect cognitive processes due to limited WM capacity. However, their findings revealed that TA did not impair performance, although their fNIRS data demonstrated that, in the lower performing group, TA (*Level 2*) was more mentally demanding. Functional near infrared spectroscopy has also been used in neuroscience research to assess the brain areas that are responsible for different cognitive processes (Pinti et al., 2015), to measure changes in mental workload (Aghajani et al., 2017) and to assess changes that are related to structural differences in the brain (Rodriguez Merzagora et al., 2014; Montgomery et al., 2017).

When considering the use of TA on endurance sports, such as cycling, it is important to consider the effects of TA on cognitive functioning and attentional focus. Rooks et al.’s (2010) systematic review considered the effects of incremental exercise on cortical oxygenation. They found that oxygenation initially increased between low and moderate intensities, remained stable for moderate to hard intensities, and then declined at maximal, exhaustive intensities. Therefore, it is possible that the concurrent reporting of thought processes when using TA may be compromised by the availability of oxygen in the cortex under higher workload. Conversely, TA may disrupt the process of increasing effort, potentially negatively affecting overall performance. This was reported by a participant during Whitehead et al.’s (2018) cycling study who commented, *“… you had to hold yourself back a little bit more to make sure you could actually speak”* (p.106). The prefrontal cortex (PFC) is considered central to WM functioning, and managing executive and attentional processes (Kane & Engle, 2002). According to the Reticular Activating Hypofrontality model (Dietrich & Audiffren, 2011), during exercise, there is decreased regulation in brain areas involved with higher-order cognition compared to regions involved with motor control. Since endurance sport performance may involve areas above VT, the competition between the PFC and brain regions responsible for movement control (the thalamus and the brain stem) creates implications for using TA during endurance sports T.

Pike et al.’s (2014) finding of TA differences in relation to performer skill levels also makes it important to consider an athlete’s experience when using TA. Higher level (more experienced) athletes may operate with different procedural structures than lower level performers, and TA may force them to verbalize an unnatural process. This is evident in endurance sports in which elite athletes are better able to resist the effects of mental fatigue, due to their superior response inhibition (Martin et al., 2016). Elite athletes’ ability to focus on relevant physical task requirements has been found to predict their performance (Cona et al., 2015). Therefore, PFC-related cognitions would appear to be an important aspect of athlete performance, perhaps especially in longer duration sporting events in which pacing may help determine success. This, in turn, could mean that trained athletes experience less interference when adopting a cognitive task during exercise performance. Further support for this hypothesis derives from the notion that well-learned skill execution becomes automated and thus requires little ongoing attention and cognitive control (Beilock et al., 2002). As such, it is reasonable to suggest that, from years of practice among higher level athletes, essential sport skills are automated, freeing up attentional resources that can be devoted to thinking aloud. Thus, one might hypothesize less reactivity in task performance from using TA for trained athletes.

In this study, we aimed to investigate the effect of TA on a self-paced cycling task performance and brain behavior among both trained and untrained participants. We predicted that trained athletes would experience no adverse performance effects or brain behavior effects from TA, whereas adverse effects would occur for untrained performers.

## Method

### Design

We implemented a mixed design with cycling expertise (untrained vs. trained) as the between groups independent variable and TA stage (5 levels) and condition (2 levels – silent vs. TA) as the within groups independent variables. Dependent variables were the oxygenation change scores in 12 areas across the PFC, and physiological indicators of % of heart rate maximum (%HRmax), blood lactate from a finger prick measurement ([La]b), rate of perceived exertion (RPE), continuous average power output of each stage (APO) and peak power output from each stage (PPO).

### Participants

We recruited participants via a social media post on Twitter, and we asked prospective participants to contact the lead author if they believed that they fit the study’s inclusion/exclusion criteria. Criteria for the trained participants stipulated that they should have a regular training week involving cycling and be currently training at least five hours and/or 60 km a week, and that they should have been training and competing in cycling events over the past three years in accordance with guidelines from prior research (De Pauw et al., 2013). Untrained participants were expected to be healthy and physically active but to have had no prior experience in competitive cycling. All participants provided written informed consent and ethical approval was granted by Liverpool John Moores University Research Ethics Committee (19/SLN/025) before the study was conducted.

We collected participants’ anthropometric data on their first visit and had them complete a short training questionnaire. Volunteers were nine cyclist-trained males (*M* age = 39, *SD* = 14 years; *M* height = 179.4, *SD* = 7.2 cm; *M* weight = 80.1. *SD* =7.4 kg; Minimum training experience = 5 × 75 minutes per week on cycling turbo sessions, road bike, swimming and running, with *M* cycling miles per week = 110, *SD* = 40) and ten physically active males (*M* = 34, *SD =*13 years*;* M height = 179.2, *SD* = 6.6 cm; *M* weight = 84.0, *SD* =17.5 kg; Minimum physical activity experience = 3 × 45 minutes per week in a mixture of football, gym, running and rowing for at least three years, with no previous experience of any structured cycling training).

## Materials

All participants performed the cycling trial on a Watt bike (Watt Bike Trainer, Nottingham). Blood lactate measurements were taken from the index finger of each participant using a small lancet to pierce the skin and we used a Lactate 2 Pro Analyzer to collect the sample. Since the intensity corresponding to the maximal equilibrium between production and removal of blood lactate has been related to aerobic performance, the use of maximal lactate steady state (MLSS) intensity to examine submaximal aerobic capacity is considered the gold standard. The results of the blood lactate finger prick at the conclusion of each stage was expected to predict the participants’ anaerobic capacity and indicate fitness (Heck et al., 1985; Beneke, 2003; Billat et al., 2003; Faude et al., 2009). Most prior research has supported using anaerobic threshold and validity, defined as the power output at [La]b of 3.5 mmol·L−1, as an indirect index of MLSS (Denadai et al., 2004, 2005; Figueira et al., 2008; Heck et al., 1985).

Participants wore a chest heart rate strap (H10 Polar) from which readings were taken at pre- and post-warm-up and at the end of each 3-minute stage. We also took participants’ post-warm-up, stage completion, and overall session ratings of perceived effort (RPE) on Borg’s (1970) 6-20 scale as per Haddad et al. (2017).

For fNIRS, we used an Oxymon III (Artinis Medical Systems, Netherlands) to collect data. We used the Oxysoft program (Artinis Medical Systems, Netherlands) for data collection, data visualization and data pre-processing. We assessed changes in oxygenated (O_2_Hb) and deoxygenated (HHb) haemoglobin in 12 areas of the PFC with transmitters and detectors fitted in to a neoprene head cap, secured with a velcro chin strap. The sampling rate was set to 50 Hz per scan, with a source-detector separation of 4.5 cm. Differential Pathway Factors were calculated based on individual participants’ ages, which ranged from 18 – 57 years old. Montage sensitivity was tested using AtlasViewerGUI for Homer2 following the process outlined in Aasted et al. (2015) (See Figure 3 for Montreal Neurological Institute (MNI) coordinates for all optodes).

A Dictaphone and a clip microphone captured TA verbalizations through the TA cycling trial only. The clip mic was clipped to the participants’ collar or cycling jersey, which was attached to a Dictaphone that was kept in the cycling jersey pocket or attached to an arm strap. However, TA data was not analyzed for this study, as it was part of a wider study and outside the aims of this study.

### Procedure

Participants were instructed to avoid any intake of caffeine or alcohol and any strenuous exercise in the 24 hours preceding a test session and to arrive at the laboratory in a rested and fully hydrated state. All tests within participants were performed at a similar time of day in a controlled environmental laboratory condition (19–22°C), to minimize the effects of diurnal biological variations. At the first session, after participants gave informed consent as noted above and had been seated for 5-minutes, we collected data for their resting blood pressure and heart rate (Dinamap V100, GE Healthcare). Their standing height (cm), body mass (kg) and training history were recorded to check that these data matched recruitment criteria. Each test was performed on a cycle ergometer with electromagnetic braking (Wattbike, Training Model, Nottingham), calibrated in accordance with the manufacturer’s guidelines, and a Wattbike performance monitor was used to collect the participant’s power, speed and cadence data. Before using the Wattbike, participants adjusted the seat height and distance from the handlebars to suit their preference, or, if they did not know a preference, we used the Wattbike User Guide set up. When participants were familiar with the bike, the fNIRS head cap was fitted and transmitter/receiver placement was adjusted as necessary. Participants were then fitted with the chest-strap HR monitor. Before commencing the trial, a 2-minute baseline was recorded for calculating the relative changes in O_2_Hb and HHb. A warm-up guide was provided, consisting of 5 minutes of steady state cycling followed by 2 × 1-minute bouts of cycling at the self-regulated pace for stage one and then for the self-regulated pace at stage two. There was then a 3-minute break until the test started.

The incremental cycling performance test consisted of five stages of three minutes of continuous cycling and one minute of active rest in between each stage to allow for participants to start steady, progress through aerobic and anaerobic threshold zones and finish on a maximal effort to be sustained for a 3-minute period (Faude et al., 2009). Participants were instructed to use the Borg Scale (Borg, 1982) to self-pace five stages of cycling and wer provided no verbal encouragement. During the warm-up, participants were familiarized with this scale and educated on each level. During each stage they were asked to keep the set self-pace consistent for the duration of each three minutes. At the end of each stage, data for average and maximum power output produced were recorded as well as physiological data involving [La]b, heart rate and RPE.

All participants engaged in two trial sessions. Participants were randomly allocated between a silent condition, in which participants were not instructed to verbalize any thoughts throughout the trials, and a TA condition. We provided detailed instructions to participants to explain the procedures involved with using the TA protocol. The TA training exercises involved using Ericsson and Simon’s (1993) adapted directions for giving TA verbal reports, which included providing verbal reports during the warm-up task and completing the following non-cycling problems: (a) an alphabet exercise, (b) counting the number of dots on a page, and (c) verbal recall. Participants were instructed to use *Level 2* TA and were asked to *“please Think Aloud by trying to say out loud anything that comes into your head throughout the trial. You do not need to try and explain your thoughts and you should speak as often as you feel comfortable in doing so.”* Based on recommendations from Birch and Whitehead (2020), participants were also asked to TA during a task specific exercise, which included thinking aloud in the laboratory-environment and task, and to TA during the warm-up. During the rest period prior to commencing the trial, participants were asked to confirm that they were fully comfortable with the task of thinking aloud, and instructions were reiterated. During the task, if participants were silent for more than 20 seconds, they were reminded to “please keep thinking aloud.” After completion of the final stage five trial, participants completed a cool down of three minutes of steady cycling.

### Data Analysis

Although we recognize the importance of an a priori power analysis to determine sample size (Schweizer & Furley, 2016), it is important to acknowledge the embryotic nature of this research. Since this is the first study of its kind, no effect size estimates were available to insert into power analysis assumptions. Thus, we conducted a post hoc power analysis using G*Power 3.1 (Faul et al. 2007) and found that, to detect a large effect size in mixed ANOVA (effect size f = .5; α = .05; groups = 2; measurements = 20; *n* = 19), our sample of 19 participants resulted in achieved power (1 – β err prob) of .81. Consequently, the current study was adequately powered. We used the Statistical Package for the Social Sciences (SPSS v25, IBM Corporation, New York, USA) to analyze all physiological, performance and fNIRS data. We set the statistical significance level at *p <* .05 for all inferential analyses.

#### Physiological data

To understand any interaction between within-subjects factor and between-subjects factor on the dependent variable a series of mixed ANOVAs with group as the between groups variable (2 levels, trained/untrained) and stage (6 levels, to also include the warm up data) as the within groups variable and changes in physiological and performance variables as the dependent variables across two conditions (Frey, 2018). Bonferroni post hoc test were used. Mauchly’s Test for Sphericity indicated a significant degree of freedom and therefore the data was adjusted accordingly using the Greenhouse-Geisser. Partial eta squared (ήp^2^) was also reported using Cohen’s guidelines with .1 being small, .3 being medium, and .5 being large (Cohen, 1988).

#### Functional Near Infrared Spectroscopy

The individual channels were visually inspected for any saturated channels and movement artefacts. A band pass filter (.01 Hz low cut off; .5 Hz high cut off) was used to remove high frequency noise and noise due to respiration, and raw data epochs for the baseline and for each stage were extracted from the continuous recording after applying the modified Beer-Lambert law logarithm in Oxysoft to calculate relative changes in cortical O_2_Hb and HHb (μmol). Correlational Based Signal Improvement (CBSI) (Cui et al., 2010) was used to reduce signal noise interference (e.g., from motion artifacts) by introducing a correction to average hemodynamic change calculations. As CBSI forces an inverse correlation between O_2_Hb and HHb, it is only necessary to report one of these parameters of cortical oxygenation after using this method. CBSI corrected O_2_Hb averages for each channel were calculated, and changes were computed relative to baseline by subtracting the CBSI average for each channel in the baseline period from each channel in each stage. fNIRS data were then analyzed using a series of mixed ANOVAs with group as the between groups variable (2 levels, trained/untrained), Condition (2 levels, Silent vs. TA) and stage (5 levels) as the within groups variables and changes in O_2_Hb at each site measured (optodes 1–12) as the dependent variables. The assumptions for ANOVA were met, and while equality of variance was not met for 10 of the 120 dependent variables (Levene’s test *p* < .05), the *n* for each group was roughly equal, so mixed ANOVA was deemed appropriate.

#### Think Aloud data

All TA data were transcribed verbatim, and transcripts ranged from 1011 words verbalized to 3013 words (m = 2256). These transcripts were analyzed as part of a separate project and are not included within this study.

## Results

We first conducted initial analyses to determine whether it would be necessary to covary for age in data analyses. As there was no significant age difference between the trained and untrained groups, *t* (18) = −1.04, *p* = .31, age was not included as a covariate factor in subsequent analyses.

### Performance Data

**Figure 1.**
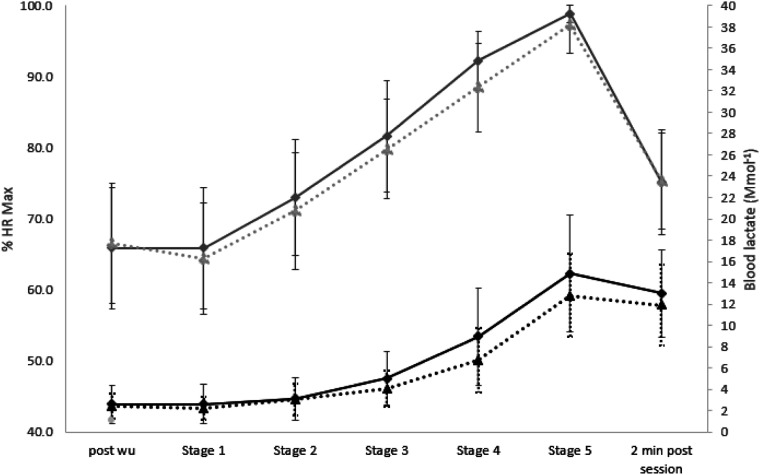
All Participants (*n* = 19) Average Percentage Heart Rate (HR%) (grey lines) and Blood Lactate ([La]b) (black lines) Responses from post Warm Up, the five Incremental Stages and Post the Final Stage Represented as the Think AlPud (dotted line) and Silent (solid line) Trial, with Standard Deviations Displayed.

Data were tested for normality using the Kolmogorov-Smirnov test; Of the 70 normality statistics computed, 48 indicated a normal distribution (*p* > .05). For the remaining 22 variables, p ranged from .001 to .049. As most variables were normality distributed and there were no extreme outliers, we used a mixed ANOVA to analyze the data. Changes in performance variables over the warm-up, five stages and two minutes’ post stage five in trained and untrained cyclists for the two conditions (TA vs. silent) are displayed in Figure 1. For the five mixed ANOVAs Mauchley’s test was significant for HR%, [La]b, APO, PPO and RPE, so Greenhouse-Geisser adjusted degrees of freedom and statistics are reported.

For HR%max, the main effect of Condition was significant, *F* (1,18) = 6.45, *p* = .02; ήp^2^ = .26, with the silent condition having a higher HR%. The Condition*Group interaction effect was also significant, *F* (1,18) = 4.59, *p* = .05 ήp^2^ = .20. There was a significant main effect of Stage, *F* (2.91, 52.34) = 115.13, *p* = .0001, ήp^2^ = .86, indicating that HR%max increased from baseline across the stages, regardless of Condition and Group. The Stage*Group interaction was, however, non-significant indicating that the groups did not differ from each other in the various stages, *F* (2.91, 52.34) = 1.29, *p* = .29. The Condition*Stage and Condition*Stage*Group interactions were also non-significant, *F* (3.48, 62.60) = 1.38, *p* = .25 and *F* (3.22, 62.60 = 1.88, *p* = .13, respectively. The effects of Group were non-significant, *F* (1,18) = .03, *p* = .88.

For [La]b performance measurements, the main effect of Condition was significant (see Figure 1), *F* (1,18) = 11.12 *p* = .004, ήp^2^ = .38. The Condition*Group interaction was non-significant, *F* (1,18) = .67, *p* = .42. There was a significant main effect of Stage, *F* (1.60, 28.82) = 96.91, *p* < .0001, ήp^2^ = .84, indicating that [La]b increased across the stages, regardless of Condition and Group. The Stage*Group interaction was, non-significant, indicating that the groups did not differ from each other in the various stages, *F* (1.60, 28.82) = 1.25, *p* = .30. The Condition*Stage interaction was significant, *F* (2.29, 41.14) = 3.50, *p* = .03 ήp^2^ = .16, meaning that the silent trial was producing more [La]b after stage 2 onwards compared to the think aloud trial. The Condition*Stage*Group interaction was non-significant, *F* (2.29, 41.14) = 1.21, *p* = .31. as was the effect of Group, *F* (1,18) = .01, *p* = .92.

For the APO performance data, the main effect of Condition was non-significant, *F* (1,18) = 3.66, *p* = .07, as was the Condition*Group interaction, *F* (1,18) = 1.45, *p* = .24. There was a significant main effect of Stage, *F* (1.56, 28.15) = 32.98, *p* < .0001, ήp^2^ = .65, indicating that APO increased across the stages, regardless of Condition and Group. The Stage*Group interaction was, non-significant indicating that the groups did not differ from each other as a function of stage, *F* (1.56, 28.14) = .28, *p* = .70. The Condition*Stage interaction was non-significant, *F* (2.17, 39.04) = 1.08, *p* = .35, and so were the Condition*Stage*Group interactions, *F* (2.17, 39.04) = .99, *p* = .39. There was a significant main effect of Group, *F* (1,18) = 6.32, *p* = .02, ήp^2^ = .26, meaning the trained cyclists APO was higher throughout.

For the PPO performance variable, the main effect of Condition was non-significant, *F* (1,18) = 1.66, *p* = .21, as was the Condition*Group interaction, *F* (1,18) = 2.68, *p* = .12. There was a significant main effect of Stage, *F* (2.32, 41.79) = 111.48, *p* < .0001, ήp^2^ = .86, indicating that PPO increased from baseline across the stages, regardless of Condition and Group. The Stage*Group interaction was, non-significant, indicating that the groups did not differ from each other in the various stages, *F* (2.32, 41.79) = 2.26, *p* = .11. The Condition*Stage and Condition*Stage*Group interactions were also non-significant, *F* (3.36, 60.49) = 1.48, *p* = .23 and *F* (3.36, 60.49) = 1.16, *p* = .33, respectively. However, in this instance, the effect of Group was significant, *F* (1,18) = 7.56, *p* = .01, ήp^2^ = .30.

**Figure 2.**
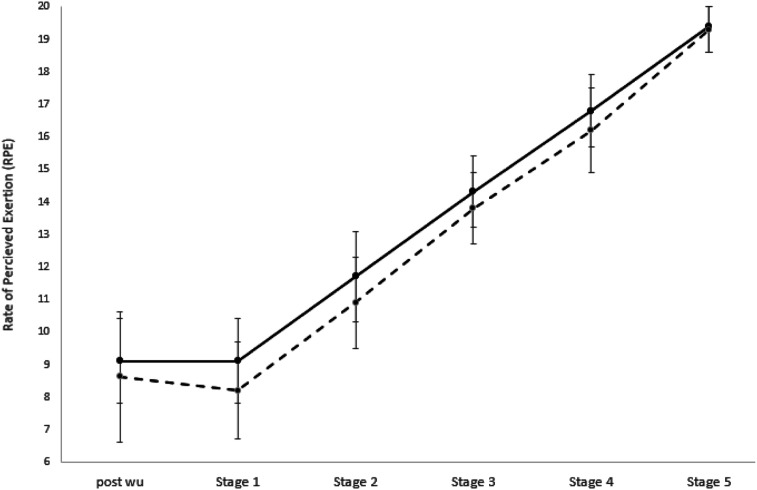
All Participants. (*n* = 19) Rate of Perceived Exertion (RPE) Responses from Post Warm Up and the Five Incremental Stage Represented as the Think Aloud (dotted line) and Silent (solid line) Trial, with Standard Deviations Displayed.

For RPE, the main effect of Condition was significant, *F* (1,18) = 18.23, *p* < .0001, ήp^2^ = .50 (Figure 2), such that the silent trial was perceived as harder over the stages. The Condition*Group interaction was non-significant, *F* (1,18) = 1.10, *p* = .31. There was a significant main effect of stage, *F* (2.21, 39.86) = 324.66, *p* < .0001, ήp^2^ = .95, indicating that RPE increased from baseline across the stages, regardless of Condition and Group. The Stage*Group interaction was non-significant, *F* (2.21, 39.86) = 1.66, *p* = .20. The Condition*Stage and Condition*Stage*Group interactions were non-significant, *F* (2.76, 49.73) = 1.18, *p* = .33 and *F* (2.76, 49.73) = .49, *p* = .68, respectively. The effects of Group were non-significant, *F* (1,18) = .90, *p* = .36.

Sessional RPE was collected at the end of each trial and participants were asked to rate how hard the session was as a whole. There was no significant difference between the responses (Silent 15 ± 2 verses TA 15 ± 2), meaning somewhat hard to hard, with *p* = .87.

### Functional Near Infrared Spectroscopy

**Figure 3.**
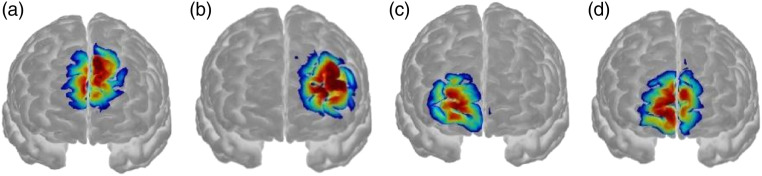
Sensitivity Profile Created using AltasViewerGUI for Homer2 as per Aasted et al. (2015) for Optodes with Significant Main Effects: Optode 4 (3a), Optode 6 (3b) Optode 8 (3c) and Optode 9 (3d). Montreal Neurological Institute (MNI) coordinates for optodes: 1 (42 59 26); 2 (18 50 23); 3 (10 53 24); 4 (−2 46 21); 5 (−12 47 20); 6 (−24 45 16); 7 (39 57 0); 8 (20 52 0); 9 (13 74 1); 10 (−4 57 4); 11 (−20 71 1); 12 (−30 61 1).

For the fNIRS data we performed 240 tests of normality using the Kolmogorov-Smirnov test, 29 were significant indicating deviation from normal distribution (ˆ<.05 in these cases ranging from .01 to .04); nonetheless mixed ANOVA was performed as 88% of the fNIRS data was normally distributed. Changes in O_2_Hb over the five stages in trained and untrained cyclists for the two conditions (TA vs. silent) are displayed in Table 1. For optodes 1, 2, 3, 5, 7, 10, 11 and 12, the main effects of Condition, Stage and Group, and the interactions between these variables were all non-significant (*p* > .05 in all cases) so these are not discussed further. For optodes 4 (left superior mid PFC), 6 (Left mid PFC), 8 (right superior PFC) and 9 (right superior mid PFC) Mauchley’s test was significant, so Greenhouse-Geisser adjusted degrees of freedom and statistics are reported. The statistics for these analyses are reported in full in Table 2, and the sensitivity profile for each optode is displayed in Figure 3. In summary, there were main effects of Stage in all optodes, with medium – large effects sizes, indicating increases in O_2_Hb as the stages progressed. The pairwise Bonferroni comparisons (see Table 2) indicated that these increases in oxygenation were particularly pronounced at optodes 8 and 9 (superior right PFC). The main effect of Condition was significant at optode 4, and the Condition*Group interaction was also significant at optode 9.

**Table 1.** Correlational Based Signal Improvement (CBSI) Corrected Cortical Oxygenation (O2Hb) Change Across the Five Stages in Each Optode Under Silent and TA Conditions.

			Silent Stages										Think Aloud Stages									
			1		2		3		4		5		1		2		3		4		5	
			M	SD	M	SD	M	SD	M	SD	M	SD	M	SD	M	SD	M	SD	M	SD	M	SD
Optode number	1	Untrained	−1.11	9.38	−2.58	9.82	4.91	9.07	7.18	11.75	.11	30.27	−.05	6.28	1.60	9.37	1.14	15.26	3.45	9.54	1.45	16.84
		Trained	−.91	4.63	.02	7.41	1.71	6.98	5.03	5.95	3.10	9.44	2.61	10.81	4.25	7.85	2.50	1.01	5.34	11.66	7.15	10.66
	2	Untrained	5.43	6.77	−5.10	20.41	2.10	12.21	5.91	8.39	1.20	12.06	1.52	2.26	.55	5.49	2.41	5.36	1.33	7.83	1.15	13.06
		Trained	.32	4.20	3.51	6.81	5.55	9.30	6.87	10.11	8.69	9.07	−.04	5.97	.57	5.57	.68	4.74	1.60	10.37	3.08	7.28
	3	Untrained	6.74	10.23	5.15	11.43	2.91	10.61	6.21	7.54	5.21	9.78	.80	1.26	−89	6.02	.26	4.69	−.40	6.66	1.04	8.02
		Trained	5.29	18.00	7.04	19.67	9.72	.45	7.03	14.54	8.01	15.33	3.06	6.47	2.20	7.09	3.26	5.70	4.90	7.87	3.46	8.92
	4	Untrained	−2.72	5.11	−3.96	4.89	−9.33	5.95	−9.93	8.93	−9.66	10.48	1.09	5.07	1.01	5.45	.51	6.17	−1.53	7.01	−3.37	6.23
		Trained	−.80	2.31	−.52	2.80	−3.37	3.63	−5.25	4.55	−6.88	6.42	−.01	5.02	−.53	6.79	02	9.92	−3.95	8.03	−5.20	5.97
	5	Untrained	1.16	11.60	4.70	21.14	6.55	26.83	5.91	33.81	8.86	27.39	5.52	17.59	10.08	22.63	11.78	27.93	11.20	26.74	11.93	24.75
		Trained	.53	6.15	2.68	4.88	.51	6.68	.54	7.88	3.49	7.27	6.90	11.36	9.88	12.28	7.26	10.36	7.98	13.77	9.17	12.54
	6	Untrained	−2.48	6.11	−5.17	6.93	−6.23	9.64	9.93	8.93	−15.41	12.66	−3.75	7.10	−4.95	9.25	−5.90	9.71	−8.18	10.42	−9.45	10.35
		Trained	−.90	3.72	−1.38	2.69	−3.19	4.14	−5.25	4.55	−6.23	6.57	−4.79	4.30	−5.00	5.33	−4.84	7.11	−5.49	3.86	−7.44	7.35
	7	Untrained	1.19	4.66	2.17	4.60	2.84	9.48	5.39	14.96	7.75	24.23	−1.59	4.99	−2.86	9.65	.64	6.56	−1.36	9.83	3.23	5.32
		Trained	.69	8.09	4.20	6.95	7.03	12.63	5.65	12.45	5.07	14.58	3.82	5.99	3.55	4.83	3.64	4.56	1.44	4.69	1.68	6.98
	8	Untrained	−5.26	6.01	−7.99	6.52	−10.51	9.13	−9.99	8.97	−8.79	9.65	−.17	4.00	.42	3.60	−1.39	4.78	−4.31	8.48	−7.35	7.83
		Trained	−.20	5.12	.75	6.37	−4.20	6.84	−5.90	7.64	−8.29	8.46	.76	3.16	−3.00	3.36	−4.05	3.60	−7.03	5.81	−9.81	8.16
	9	Untrained	2.43	2.13	4.37	3.12	6.00	2.60	8.73	2.10	14.28	8.78	1.79	2.58	2.60	1.95	3.49	2.79	4.27	3.24	5.07	6.54
		Trained	.52	1.93	2.09	2.25	2.13	3.19	4.09	3.32	7.64	2.95	1.26	3.29	2.36	5.29	3.05	5.17	5.52	6.10	8.28	6.25
	10	Untrained	−1.72	3.94	−5.65	6.44	−5.36	9.35	−.49	26.81	−5.89	27.41	−.8/0	8.05	.95	11.43	.15	15.73	−.53	11.27	4.97	22.73
		Trained	−.05	3.54	−1.67	2.49	−2.28	3.11	−9.98	16.15	−8.72	5.28	−1.42	12.36	−.70	19.67	−2.51	15.73	−8.85	13.35	−12.16	10.60
	11	Untrained	4.45	9.98	−4.87	14.57	−7.44	24.96	−3.76	24.79	2.99	31.59	10.77	27.23	5.39	20.02	9.81	19.09	9.23	17.58	8.70	13.33
		Trained	−4.88	14.57	1.58	6.61	−1.93	19.88	4.94	11.42	−5.12	15.48	7.85	19.19	4.40	22.04	7.92	19.64	3.05	15.74	2.51	19.91
	12	Untrained	−.001	.004	−.001	.003	−.001	.003	−.001	.031	−.007	.003	.001	.001	.003	.005	.001	.003	.006	.001	.001	.001
		Trained	.001	.001	.011	.001	.001	.001	.001	.001	.001	.001	.001	.001	.001	.001	.001	.001	.001	.001	.001	.001

**Table 2.** Mixed ANOVA Statistics and Significance Levels for Optodes with significant Main Effects.

	Conditio*n* (1.17)		Condition*Group (1.17)		Stage					Stage*Group		Condition*Stage					Condition*Stage*Group			Group (1.17)	
	F	p	F	p	df	F	p	Ƞp^2^	Significant Pairwise Comparisons	df	F	p	df	F	p	Ƞp2	df	F	p	F	p
Optode 4	4.64	.05	1.94	.18	(2.14.31.18)	11.37	.001	.40	Stage 1 and 4 – *p* = .002 Stage 1 and 5 – *p* = .009 Stage 2 and 4 – *p* = .001	(2.14.31.18)	.41	.68	(1.83.31.18)	1.68	.20	—	(1.83.31.18)	.34	.70	.69	.42
Left superior mid PFC																					
Optode 6	.03	.86	70	.41	(2.15.42.34)	8.02	.001	.32	Stage 1 and 4 – *p* = .04 Stage 1 and 5 – *p* = .03 Stage 2 and 5 – *p* = .04	(2.1542.34)	.1.17	.33	(2.49.42.34)	1.80	.17	—	(2.49.42.34)	.55	.62	2.12	.16
Left mid PFC																					
Optode 8	1.78	.20	3.68	.07	(2.79.43.07)	13.62	.001	.45	Stage 1 and 3 – *p* = .04 Stage 1 and 4 – *p* = .005 Stage 1 and 5 – *p* = .001 Stage 2 and 4 – *p* = .04 Stage 2 and 5 – *p* = .002	(2.79.43.07)	.74	.53	(2.53.43.07)	1.83	.16	—	(2.53.43.07)	1.41	.25	.42	.53
Right superior PFC																					
Optode 9	2.36	.14	5.65	.03	(1.63.28.55)	25.07	.0001	.60	Stage 1 and 2 – *p* = .02 Stage 1 and 3 – *p* = .006 Stage 4 and 1 – *p* = .0001 Stage 4 and 2 – *p* = .0001 Stage 4 and 3 – *p* = .0001 Stage 5 and 1 – *p* = .0001 Stage 5 and 2 – *p* = .001 Stage 5 and 3 – *p* = .005	(4.63.28.55)	.11	.85	(1.68.28.55)	3.35	.06	—	(1.68.28.55)	3.54	.05	2.27	.14
Right superior mid PFC

## Discussion

The aim of this study was to investigate the effect of TA on performance and brain oxygenation in both trained and untrained participants during a self-paced cycling trial. We predicted that, for trained athletes, TA would have no effect on performance and brain oxygenation, whereas there would be opposite findings for untrained cyclists. However, we found no significant differences between groups for changes in brain oxygenation, even though performance variables for the trained participants demonstrated higher APO and PPO across the incremental exercise. Irrespective of Group and Condition, there were changes in oxygenation as the stages progressed, indicating increases in cortical oxygenation.

When examining whole group comparisons for Condition (silent vs. TA), there were significant differences between HR% max and blood lactate measurements, with the silent trial producing higher heart rates and greater blood lactates; however, there was no significant condition difference on performance variables of APO and PPO. This finding has also been evident in previous research (e.g., Whitehead et al., 2015; Fox et al., 2011) in that *Level 2* TA verbalization does not disrupt performance outcomes. However, the current study made a novel contribution in that while previous research has been conducted on self-paced motor skill tasks such as golf (Whitehead et al., 2015) and on complex problem solving tasks (Gagne & Smith, 1962; Fox et al., 2011), we investigated the effects of TA on closed skill endurance performance. As the participants’ RPE were higher in the silent compared to the TA trial throughout, with no differences in PPO and APO, there is evidence here of more efficiency in pacing the effort with help from TA. This inference is further corroborated by a an internal physiological finding of lower blood lactate and HRmax% throughout the TA trials when compared the silent trials. Thus, TA seems to assist more autonomous self-regulation of effort and pace, meaning the participant is consciously thinking more about maintaining a realistic pace, instead of thinking “about nothing” during each three-minute stage making the effort “more manageable.” Moreover, within the power output performance data there are higher values produced by the trained athletes compared to the untrained group although no difference is seen between the trials (TA vs. Silent) or between the increments of power outputs both average and peak, within the stages and within each group. Trained athletes demonstrate higher performance outcomes in APO and PPO, with similar HR% and [La]b values to the untrained, meaning the trained group have a larger range of values from steady state to maximum, demonstrating a higher level of aerobic capacity (fitness).

Most of our comparisons on fNIRS measures were non-significant, with the exception of the effects of Stage, in optodes 4, 6, 8 and 9, the effects of Condition at optode 4, and the Condition*Group and Condition*Stage*Group interactions at optode 9. Thus for the majority of sites measured, TA did not affect changes in cortical hemodynamics. Significant main effects of Stage at optodes 4, 6 and 8 indicated that oxygenation *decreased* from baseline over the five stages; given the inverse relationship between O_2_Hb and HHb, it can be assumed that this would indicate an increase in HHb. Increases in HHb are observed where there is an increase in oxygen consumption in a brain region (Obrig & Villringer, 2003), and this increase in oxygenation consumption is indicative of an increase in cognitive demand/monitoring requiring areas of the PFC over the 5 stages (e.g., Funahashi, 2017; Montgomery et al., 2017; Roberts & Montgomery, 2015). In optode 9, the significant main effect of Stage reflects increases in glucose and oxygen utilization in the PFC as the stages progressed. Inspection of the mean O_2_Hb changes in Table 1 suggests that, paradoxically, the significant Condition*Group interaction at optode 9 (right mid PFC) is due to lower increases in O_2_Hb during the TA condition than the silent condition in trained versus untrained cyclists. Table 2 also shows that, for this optode, the effect of Stage was highly significant, with O_2_Hb changes in stages 4 and 5 differing significantly from all other stages; we suggest that the significant Condition*Group interaction here should be treated with caution as it could be an artifact of the highly significant effects of Stage. It is also possible that the during the TA condition, the left PFC is involved in supporting articulation of exercise cognitions, and thus resources are diverted from the right PFC, resulting in the significant effect of Condition in optode 4 and the significant Condition*Group interaction in optode 9. Future research should specifically investigate the relative roles of the right and left medial PFC in supporting TA during physical activity. Although previous research suggests that using TA during the completion of a task, may disrupt or alter cortical hemodynamics in novice participants (Pike et al., 2014), our findings suggest that using TA does not adversely affect performance as measured by changes in cortical hemodynamics. In addition, at the intensities used in the current protocol, participants were able to use TA without a significant increase in cortical demand. However, it is important to note that although our active participants were novice cyclists, they were physically active, and, therefore, some level of transferability across sports could have occurred. Further studies may consider using novices who are inactive and have near to no experience of sport or physical activity.

### Limitations and Directions for Further Research

It is important to note the limitations of this study. Since this is the first study of its kind, no effect size estimates were available to insert into a priori power analysis assumptions. Thus, we conducted a post hoc power analysis which revealed that the study was adequately powered. But as some effects approached significance, a larger sample size would have allowed us to make more robust interpretations of these trends and would have more safely permitted generalization to other populations. Nonetheless, this study provides important implications for future researchers when considering the use of the TA method and when capturing cognition data in endurance activity. We argue that this is a significant contribution of this manuscript. Future researchers should not only consider larger sample sizes, but potentially a wider range of participant expertise. Furthermore, given that our study included a participant sample with a wide age range, we recommend that future investigators recruit certain age cohorts to better control for potential age effects. In addition, although we used De Pauw et al. (2013) criteria for our trained group, we did not collect exact means and standard deviations of previous training times within each group. By collecting this in future work, researchers can better infer differences between a wider range of experience performers.

Also when considering directions for future research, we did not study the quality and completeness of the TA verbalizations as participants reached the higher intensity interval stages and VT. If oxygenation declines at maximal, exhaustive intensities (VT) (Rooks et al., 2010), it is possible that the concurrent report of thought processes via TA may become compromised, incomplete or distorted by the reduced availability of oxygen in the cortical areas of the brain under higher workload. Although we can confirm that TA occurred throughout all stages of the five interval trials, future investigators should consider the content of this TA data across different work load intensities and also understand the blood flow distribution from both areas within the brain and the working muscles. Although we were able to investigate PFC through fNIRS, we have not yet developed an understanding of how blood flow distributions and amount are prioritized through vascular shunting from areas of the brain to cope with the demands of the exercise task. Future researchers might use a transcranial Doppler at rest and during the task to assess these blood flow changes in addition to measuring relative changes in cortical oxygenation.

## Conclusion

Although previous researchers have suggested that TA might disrupt task, we demonstrated that TA use during an incremental self-paced cycling test to maximum effort resulted in no significant performance decrements when compared to a silent trial. In addition, changes in cortical hemodynamics were only evident in one area as a function of TA versus silent conditions, indicating that TA, on the whole, does not require additional resources above what is required during the performance of this trial. In the context of limitations highlighted in our discussion, this study has advanced TA research by providing initial evidence that TA does not disrupt performance outcomes at low through to high levels of physical exertion in either untrained or trained participants. In addition, from a practical perspective, if coaches or sport psychologists wish to further understand their athletes’ thought processes during performance, they might worry less about performance disruption associated with TA use.
